# Cardiotoxicity in patients with metastatic melanoma treated with BRAF/MEK inhibitors: a real-world analysis of incidence, risk factors, and reversibility

**DOI:** 10.2340/1651-226X.2025.42567

**Published:** 2025-04-13

**Authors:** Jonas K. Oddershede, Ida K. Meklenborg, Lars Bastholt, Louise M. Guldbrandt, Henrik Schmidt, Rasmus B. Friis

**Affiliations:** a Department of Oncology, Aarhus University Hospital, Aarhus, Denmark; b Department of Oncology, Odense University Hospital, Odense, Denmark

**Keywords:** BRAF/MEK inhibition, cardiotoxicity, malignant melanoma, echocardiography, multigated acquisition scan, oncocardiology, antineoplastic therapy

## Abstract

**Background:**

BRAF/MEK inhibitors (BRAFi/MEKi) improve outcome in patients with BRAF-mutated metastatic melanoma but are associated with cardiotoxicity, leading to a decline in left ventricular ejection fraction (LVEF). This study aimed to evaluate the incidence, timeline, risk factors, and reversibility of BRAFi/MEKi-induced cardiotoxicity in a real-world setting.

**Patients/materials and methods:**

Patients with metastatic melanoma (*n* = 170) treated with Encorafenib/Binimetinib, Vemurafenib/Cobimetinib, or Dabrafenib/Trametinib at Aarhus and Odense University Hospital, Denmark, from 2015 to 2023 were included. Cardiac function was assessed at baseline and every 3 months during treatment with either echocardiograms or multigated acquisition scans. Cardiotoxicity was defined as a reduction of LVEF by ≥10 percentage points (pp) to an LVEF < 50% (*Major cardiotoxicity*) or a reduction of LVEF by ≥15 pp but remaining > 50% (*Minor cardiotoxicity*).

**Results:**

Cardiotoxicity occurred in 21% of patients, with 14% experiencing major cardiotoxicity. The mean time to LVEF decline was 187 days, with 92% of major cardiotoxicity cases occurring within the first year. Cardiotoxicity was reversible in 79% of patients following dose reduction, treatment pauses, heart failure therapy, or continued treatment with monitoring. Baseline atrial fibrillation (odds ratio 13.67, *p* = 0.008) was identified as a risk factor for major cardiotoxicity.

**Interpretation:**

BRAFi/MEKi-induced cardiotoxicity is a significant but manageable complication, often reversible with timely interventions. Routine LVEF monitoring is recommended. The majority (92%) of major cardiac events were diagnosed within the first year of treatment, which might warrant a discontinuation of routine LVEF monitoring after 1 year of BRAFi/MEKi treatment.

## Introduction

Melanoma is a highly aggressive and potentially lethal form of skin cancer causing approximately 55,500 cancer-related deaths annually worldwide, with steadily increasing incidence, making it a significant public health concern [1, 2]. The molecular landscape of melanoma is characterized by a diverse array of genetic alterations, including mutations in the BRAF gene present in about 50% of melanomas [3]. BRAF(V600)-inhibitors (BRAFi) – such as Dabrafenib, Encorafenib, and Vemurafenib – target mitogen-activated protein kinase (MAPK) signaling pathway in melanocytes with evidence to improve progression-free survival (PFS) and overall survival (OS) [4, 5]. MEK is a kinase enzyme downstream to RAF that may be constitutively active in up to 8% of melanomas [6] and can be targeted with MEK inhibitors (MEKi) – such as Trametinib, Binimetinib, and Cobimetinib. Clinical trials have shown that the combination of BRAFi and MEKi improves PFS and OS compared to BRAFi alone with diminished side effects [7–12].

BRAFi can cause modest QT interval prolongation and are associated with a mild left ventricular ejection fraction (LVEF) reduction in 2–5% of patients [13]. In contrast, MEKi have a more pronounced cardiotoxic effect, significantly increasing the risk of LVEF decline. Combination therapy further amplifies this risk, leading to a 3-fold higher likelihood of LVEF reduction (relative risk [RR]: 3.72; 95% confidence interval [CI]: 1.74–7.95) [13, 14].

The clinical manifestations vary, ranging from asymptomatic changes in cardiac function to life-threatening events such as heart failure and arrhythmias [15, 16]. Efforts to mitigate the risk of cardiotoxicity associated with BRAFi/MEKi therapy have focused on close monitoring of cardiac function using imaging modalities such as echocardiograms (ECHO) or multiple-gated acquisition (MUGA) scans. For the individual patient, dose modifications, interruption, or discontinuation of BRAFi/MEKi may be necessary to manage cardiotoxicity [17–19]. On the contrary, considering the continuously rising healthcare costs, it becomes crucial to accurately identify, assess, and manage BRAFi/MEKi-induced cardiotoxicity, including associated risk factors. Such evaluations not only guide clinical decision-making and enhance patient outcomes but may also potentially reduce the necessity for prolonged and frequent utilization of cardiac imaging modalities [20].

The primary objectives of this study were to identify the incidence of BRAFi/MEKi-induced cardiotoxicity and evaluate the time to reduction in LVEF including risk factors and reversibility.

## Patients/materials and methods

### Study population

All patients receiving Encorafenib/Binimetinib, Vemurafenib/Cobimetinib, or Dabrafenib/Trametinib at the Department of Oncology, Aarhus University Hospital, Denmark, and the Department of Oncology, Odense University Hospital, Denmark, were identified since the approval of MEKi in 2015 until data cutoff June 2023.

The inclusion criteria included patients with a diagnosis of unresectable stages III–IV melanoma, who underwent treatment with Encorafenib/Binimetinib, Vemurafenib/Cobimetinib or Dabrafenib/Trametinib. Additionally, patients were required to have at least two available MUGA-scans or ECHO, with the first scan conducted at baseline or within 1 week after the start of BRAFi/MEKi treatment, and the second scan approximately 3 months after therapy initiation. Furthermore, follow-up MUGA-scans or ECHO were routinely performed at least every 3 months during the course of treatment.

Exclusion criteria encompassed patients who did not have a baseline MUGA-scan or ECHO, for example, due to an urgent requirement for therapy. Additionally, patients with either no or only one MUGA-scan or ECHO were excluded, such as those who did not undergo the initial evaluation due to disease progression or death. BRAFi-monotherapy-treated patients were excluded due to inconsistent monitoring and minimal cardiotoxicity risk [13].

In the Danish Metastatic Melanoma Database (DAMMED), clinical data are registered on all Danish melanoma patients receiving systemic antineoplastic therapy [21]. Informed consent is signed by all patients registered in DAMMED. The registry and consent form have received legal approval from the Danish Data Protection Agency (18/47885) and the Danish Patient Safety Authority (3-3013-1688/1/).

### Evaluation of cardiac function

LVEF served as the primary metric for evaluating cardiac function and was retrospectively assessed by reviewing patient medical records. LVEF measurements were obtained through either MUGA-scans or ECHO, conducted by a medical doctor/specialist nurse at the time of assessment, and subsequently documented in the patient records.

### Cardiotoxicity

Cardiotoxicity was defined as a decrease in LVEF to less than 50% or a reduction in LVEF greater than 15 percentage points (pp) [22, 23]. A decline of more than 10 pp to an LVEF below 50% was classified as *major cardiotoxicity*, while a decrease in LVEF of more than 15 pp, but still above 50%, was considered *minor cardiotoxicity*. Patients with an LVEF below 50% at baseline were analyzed separately. Cases exhibiting cardiotoxicity were further assessed for reversibility. *Full reversibility* was defined as the restoration of LVEF to a value within 5 pp of baseline, *partial reversibility* indicated LVEF improvement by at least 10 pp but still remaining more than 5 pp below baseline, and *no reversibility* denoted LVEF improvement of less than 10 pp and remaining more than 5 pp below baseline [24, 25].

### Additional variables

Relevant patient-, disease-, and treatment-related variables, which could potentially impact the response to BRAFi/MEKi, were extracted from DAMMED. Patient records were examined to identify variables associated with cardiovascular disease, such as hypertension, hypercholesterolemia, heart failure, atrial fibrillation, diabetes mellitus, chronic obstructive lung disease (COLD), smoking, and history of other cancers [26]. Comorbidities were included if the patient was receiving medical treatment for the respective condition when initiating BRAFi/MEKi.

Electrocardiograms (ECGs) at baseline were assessed as either sinus rhythm or abnormal (prolonged PR-interval/AV-block (atrioventricular-block), bundle-branch block (right/left/complete/partial), QTc-elongation, T-inversion or arrythmia).

### Statistical analysis

Continuous variables were expressed as means ± standard deviation, and categorical variables were expressed as numbers and percentages. Group comparisons on baseline characteristics were performed with the Fisher’s exact test for categorical variables and with non-parametric *t*-test (Kolmogorov–Smirnov) when appropriate for continuous variables. A Receiver Operating Characteristic (ROC) analysis was conducted for variables requiring assessment of their predictive capability concerning the development of cardiotoxicity. Univariate survival analyses regarding cardiotoxicity, OS, and PFS were carried out on discontinuous variables with the Mantel-Cox Log-Rank test. A *p*-value < 0.05 was considered significant. All calculations and plots were performed using GraphPad Prism version 10.

## Results

### Study population

**Figure 1:**
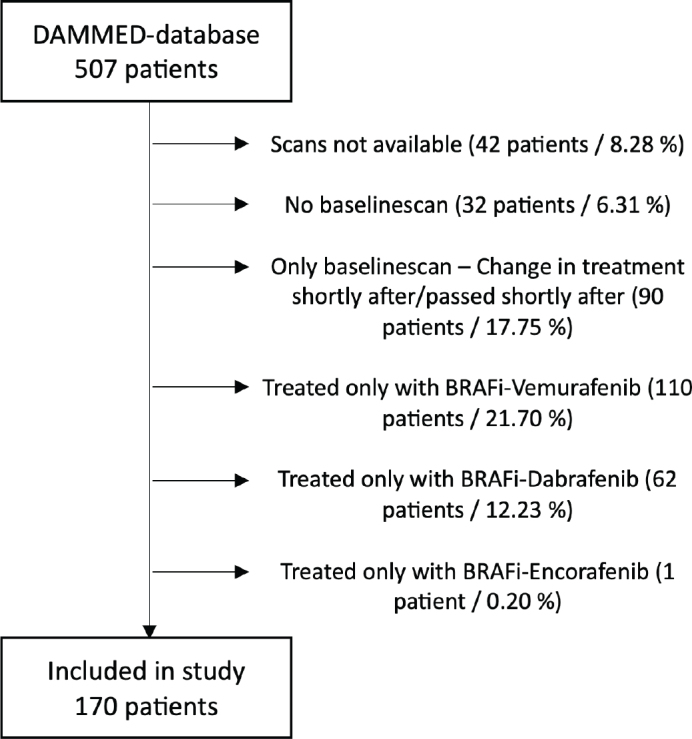
Exclusion flowchart. Patients treated with BRAFi/MEKi identified from the DAMMED-database and reason for exclusion. DAMMED: Danish Metastatic Melanoma Database; BRAFi/MEKi: BRAF-inhibitor/MEK-inhibitor.

In total, 507 patients were retrospectively identified being treated with BRAFi/MEKi. Of these, 170 patients (97 men, mean age 63.5 ± 12.9 years) were included in the study – Exclusion flowchart, Figure 1. Baseline characteristics are summarized in Table 1. The overall incidence of cardiotoxicity among patients receiving BRAFi/MEKi combination therapy was 21%. Major cardiotoxic events occurred in 14% of patients, while minor cardiotoxic events were observed in 7%. In our study, the median duration of BRAFi/MEKi treatment was 472 days (interquartile range: 398–731.75 days), and the median follow-up time was 633 days (interquartile range: 580.5–1058.5 days). A total of 885 scans were identified with a mean number of 5.2 scans per patient. Of these, 170 were baseline scans, 170 were performed approximately 3 months after baseline, and 542 were carried out for subsequent evaluations.

**Table 1:** Baseline characteristics.

Baseline characteristics	All patients	Patients with no LVEF decline	Patients with minor cardiotoxicity	*p*-value (minor vs. no)	Patients with major cardiotoxicity	*p*-value (major vs. no)
Number of patients	170	134 (79)	12 (7)		24 (14)	
Gender, male	97 (57)	71 (53)	8 (67)	0.5472	18 (75)	0.0722
Age (mean ± SD) at baseline	63.5 ± 12.9	62.9 ± 13.2	63.2 ± 11.8	1	66.7 ± 11.3	0.2866
Image modality						
- ECHO	76 (45)	66 (49)	3 (25)	0.1366	7 (29)	0.0787
- MUGA	94 (55)	68 (51)	9 (75)		17 (71)	
Performance status						
- 0–1	131 (77)	103 (77)	11 (92)	0.4645	17 (71)	0.6044
- ≥ 2	39 (23)	31 (23)	1 (8)		7 (29)	
Metastatic disease status						
- Stage M1a/M1b	47 (28)	37 (28)	4 (33)	0.6440	6 (25)	0.8503
- Stage M1c	70 (41)	55 (41)	6 (50)		9 (38)	
- Stage M1d	53 (31)	42 (31)	2 (17)		9 (38)	
BRAF-mutation						
- V600E	145 (85)	117 (87)	8 (67)	0.1082	20 (83)	0.6481
- V600K	19 (11)	12 (9)	4 (33)		3 (13)	
- V600R	2 (1)	2 (1)	0 (0)		0 (0)	
- Other	4 (2)	3 (2)	0 (0)		1 (4)	
pLDH						
- < ULN	72 (42)	60 (45)	4 (33)	0.5512	8 (33)	0.3729
- > ULN	98 (58)	74 (55)	8 (67)		16 (67)	
pAlbumin						
- > LLN	141 (83)	112 (84)	10 (83)	1	19 (79)	0.5650
- < LLN	29 (17)	22 (16)	2 (17)		5 (21)	
BRAFi/MEKi no. line of treatment						
- 1^st^ line of treatment	61 (36)	43 (32)	4 (33)	0.1860	14 (58)	0.0042*
- 2^nd^ line of treatment	82 (48)	69 (51)	6 (50)		7 (29)	
- 3^rd^ line of treatment	23 (14)	19 (14)	2 (17)		2 (8)	
- 4^th^ line of treatment	3 (2)	2 (1)	0 (0)		1 (4)	
- 5^th^ line of treatment	0 (0)	0 (0)	0 (0)		0 (0)	
BRAFi/MEKi-subtype						
- Encorafenib/Binimetinib	77 (45)	69 (51)	3 (25)	1	5 (21)	0.1548
- Dabrafenib/Trametinib	92 (54)	64 (48)	9 (75)		19 (79)	
- Vemurafenib/Cobimetinib	1 (1)	1 (1)	0 (0)		0 (0)	
Previous cancer	30 (18)	24 (18)	4 (33)	0.2451	3 (13)	0.7687
Comorbidities						
- Hypertension	63 (37)	47 (35)	6 (50)	0.3538	10 (42)	0.6451
- Hypercholesterolemia	44 (26)	34 (25)	2 (17)	0.7306	8 (33)	0.4547
- Ischemic heart disease	9 (5)	6 (4)	1 (8)	0.4586	2 (8)	0.3494
- Atrial fibrillation	21 (12)	11 (8)	2 (17)	0.2898	8 (33)	0.0022*
- Diabetes mellitus	30 (18)	21 (16)	1 (8)	0.6938	8 (33)	0.0487*
- COLD	11 (6)	9 (7)	0 (0)	1	2 (8)	0.6744
Smoking (previous/current)	76 (45)	61 (46)	5 (42)	1	10 (42)	0.8252
Abnormal ECG	31 (18)	21 (16)	2 (17)	1	8 (33)	0.0487*
LVEF (%) at baseline	59.8 ± 8.2	59.0 ± 7.9	70.1 ± 8.9	< 0.0001*	58.9 ± 5.4	0.9201

Baseline characteristics of the study population with statistical analysis of patients with minor and major cardiotoxicity, compared to patients with no decline in LVEF. Continuous variable expressed as means ± standard deviation (SD), and categorial variables expressed as numbers and percentages.

* *p* < 0.05.

SD: Standard deviation; ECHO: Echocardiogram; MUGA: multiple-gated acquisition; ULN: Upper limit of normal; LLN: Lower limit of normal; pLDH: plasma lactate dehydrogenase; pAlbumin: Plasma albumin; BRAFi/MEKi: BRAF and MEK inhibitor; COLD: chronic obstructive lung disease; ECG: Electrocardiogram; LVEF: Left ventricular ejection fraction.

### Time to cardiotoxicity

**Figure 2:**
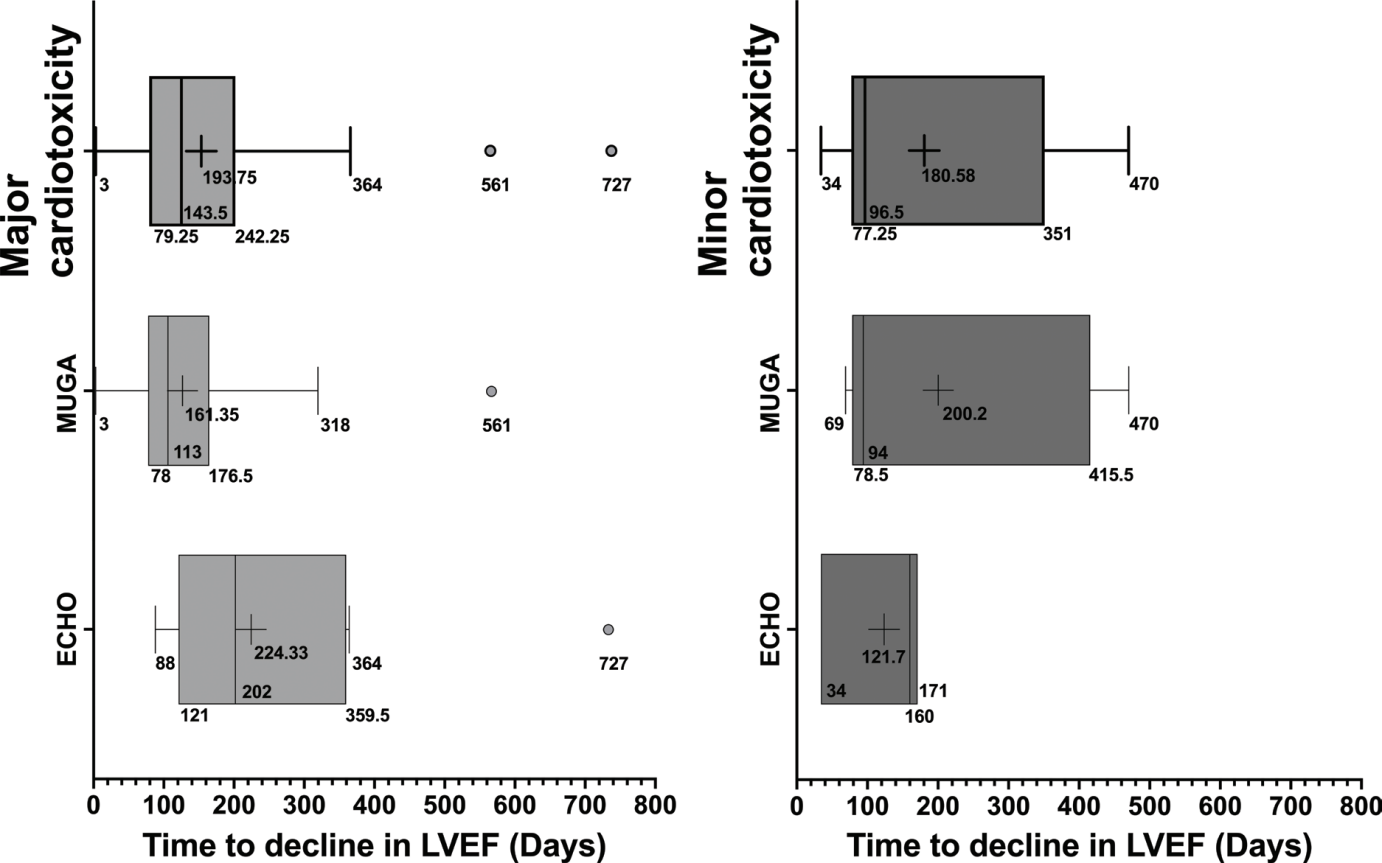
Boxplot – Time to cardiotoxicity. Range and average time to cardiotoxicity in days from the initiation of BRAFi/MEKi for all patients and stratified by the imaging modality used for monitoring. Data represented with boxplot including lower and upper extreme (whiskers) with outliers, lower and upper quartile (25^th^ and 75^th^ percentile), median (50^th^ percentile), and mean (cross). MUGA: multiple-gated acquisition; ECHO: Echocardiogram; LVEF: Left ventricular ejection fraction.

The median time to decline in LVEF was 124.5 days for all patients (Table 2, Figure 2). 92% of patients with major cardiotoxicity experienced a decline in LVEF before the 1-year evaluation with two outliers with later declines at 18.7 and 24.2 months, after BRAFi/MEKi-therapy initiation. The patient who exhibited a decline in LVEF after 18.7 months was assessed via MUGA scan, which was deemed erroneous. A follow-up echocardiography revealed a normal LVEF above 50%. The patient remained asymptomatic, and no changes in treatment were made. The patient who experienced a decline after 24.2 months had preexisting COLD. Over a 2-year period, there was a gradual worsening of COLD, which correlated with a progressive decline in LVEF. Subsequent improvement in COLD corresponded with a complete reversal of the LVEF decline, without any modifications to BRAFi/MEKi treatment.

**Table 2:** Median time to decline including range.

Patient group	Median time to decline in LVEF (days)	Range (days)
All patients with cardiotoxicity	124.5	3–727
Minor cardiotoxicity	143.5	34–470
Major cardiotoxicity	96.5	3–727

Range and median time to cardiotoxicity in days from the initiation of BRAFi/MEKi for all patients.

LVEF: Left ventricular ejection fraction.

Of the patients who experienced major cardiotoxicity, 29% were monitored with echocardiography, while 71% were followed using MUGA-scans. No significant difference in the detection time of cardiotoxicity onset was observed between the two imaging modalities (Figure 2).

Among all the patients with minor cardiotoxicity, all patients experienced a decline in LVEF before the 470^th^ day after BRAFi/MEKi-therapy initiation. The median time to decline in LVEF was 96.5 and 143.5 days for major and minor cardiotoxicity, respectively. Of the patients who experienced minor cardiotoxicity, 25% were monitored with echocardiography, while 75% were followed using MUGA-scans. No significant difference in the detection time of cardiotoxicity onset was observed between the two imaging modalities (Figure 2).

### Pattern of cardiotoxicity

#### Minor cardiotoxicity

The univariate analysis of baseline characteristics revealed that patients who developed minor cardiotoxicity had significantly higher baseline LVEF compared to others. None of the patients who developed minor cardiotoxicity developed symptoms of heart failure, initiated heart failure treatment, and underwent dose-reduction or treatment pause. All resumed MEKi therapy without further reduction in LVEF.

#### Major cardiotoxicity

For 10 patients (41%) experiencing major cardiotoxicity, the decline in LVEF was fully or partially reversible after treatment alterations. Of these 10 patients, three patients had full reversibility after dose-reduction. Five patients showed reversibility after treatment pause. One patient showed reversibility after the start of heart failure treatment and continuation of BRAFi/MEKi-therapy, and one patient showed reversibility after treatment pause combined with heart failure treatment. Three patients (13%) died shortly after treatment pause because of disease progression.

Eleven patients (46%) had no alterations in treatment and were continually monitored with MUGA-scans or ECHO every third months as they showed little or no symptoms of heart failure. Among these 11 patients, five patients had spontaneous full reversibility, and four patients had partial reversibility during continued BRAFi/MEKi treatment. Two patients had no reversibility.

In summary, the decline in LVEF was fully or partially reversible in 19 patients (79%), following interventions such as dose reduction, treatment pause, initiation of heart failure therapy, or continuous monitoring of LVEF without any changes in treatment.

In total, 41 patients had reintroduction of BRAFi/MEKi-therapy, of which four patients (10%) experienced major cardiotoxicity. Of the four patients, two experienced major cardiotoxicity during both the first and second cycles of treatment. One patient experienced major cardiotoxicity during the first cycle but showed no signs of cardiotoxicity after reintroduction with the same dosage-regimen. The remaining patient showed no cardiotoxicity during the initial treatment round but developed major cardiotoxicity after reintroduction.

### Patients with LVEF < 50% at baseline

Seven patients were identified having an LVEF < 50% at baseline. Four patients had borderline reduced LVEF to 45% and had no symptoms or received treatment for heart failure. These patients received normal dosage of BRAFi/MEKi at initiation. Three of these four patients had stable LVEF throughout the treatment, and one experienced a significant decline of LVEF to 30%. These patients were pooled in Table 1, and the multivariate analysis as ‘no decline’ and ‘major cardiotoxicity’.

Three patients had a significant lower LVEF at baseline (< 25%) and treated with an initial 50% dose-reduction. Of these, one patient received treatment for heart failure at the time of treatment initiation but died shortly after the follow-up scan due to disease progression. Two patients had no change in LVEF during treatment.

### Risk factors

In the univariate analysis, we found a significant correlation between the development of major cardiotoxicity and the presence of atrial fibrillation at baseline. A significant correlation was also shown for abnormal ECG at baseline, patients with Diabetes Mellitus, and for patients who had received other antineoplastic therapy (eg. chemotherapy, immunotherapy) prior to BRAFi/MEKi-therapy.

To identify independent predictors of cardiotoxicity and to control for potential confounding, a multiple logistics regression model was performed (Table 3). The ROC-curve as a predictor of major cardiotoxicity has an Area Under the Curve (AUC) of 0.850 (standard error [SE] = ± 0.051, *p* < 0.0001) (ROC-curve – Supplementary figure 1).

**Table 3:** Multiple logistic regression model for major cardiotoxicity depending on baseline characteristics.

Clinical variable	OR (95% CI)	*p*
Gender (male)	2.35 (0.70–7.90)	0.169
Age at first diagnosis	1.08 (0.97–1.19)	0.169
Age at baseline	0.94 (0.84–1.05)	0.258
Metastatic disease, stage m1c/m1d	0.81 (0.22–3.04)	0.754
BRAF-mutation, V600E	0.90 (0.21–3.04)	0.887
Performance status ≥ 2	0.90 (0.25–3.28)	0.870
Smoking status, prior/current	1.20 (0.37–3.92)	0.766
ECG-status, abnormal	0.75 (0.13–4.30)	0.747
pLDH > ULN	0.97 (0.30–3.10)	0.955
pAlbumin < LLN	1.33 (0.33–5.36)	0.685
Hypertension	0.70 (0.18–2.70)	0.599
Hypercholesterolemia	0.93 (0.22–3.93)	0.917
Ischemic heart disease	0.85 (0.09–7.84)	0.883
Atrial fibrillation	13.67 (1.96–95.51)	0.008*
Diabetes mellitus	2.71 (0.77–9.56)	0.121
COLD	2.31 (0.27–20.03)	0.447
Previous cancer	0.28 (0.05–1.63)	0.145
BRAFi/MEKi as > 1^st^ line of treatment	0.48 (0.14–1.69)	0.254
BRAFi/MEKi-type, Encorafenib/Binimetinib	0.17 (0.04–0.69)	0.013*
LVEF af baseline	0.98 (0.91–1.05)	0.609

Multiple logistic regression analysis for major cardiotoxicity depending on baseline characteristics.

OR: Odds ratio; CI: Confidence interval;

* *p* < 0.05; ECG: Electrocardiogram; pLDH: Plasma Lactate dehydrogenase; ULN: Upper limit of normal; LLN: Lower limit of normal; pAlbumin: Plasma albumin; COLD: chronic obstructive lung disease; BRAFi/MEKi: BRAF and MEK inhibitor; LVEF: Left ventricular ejection fraction.

The multiple logistic regression model indicates that baseline atrial fibrillation is associated with an increased risk of major cardiotoxicity, while the use of Encorafenib/Binimetinib is linked to a reduced risk of major cardiotoxicity.

In the univariate analysis of baseline characteristics, a significant correlation was also observed between high baseline LVEF and minor cardiotoxicity.

### OS and PFS

**Figure 3:**
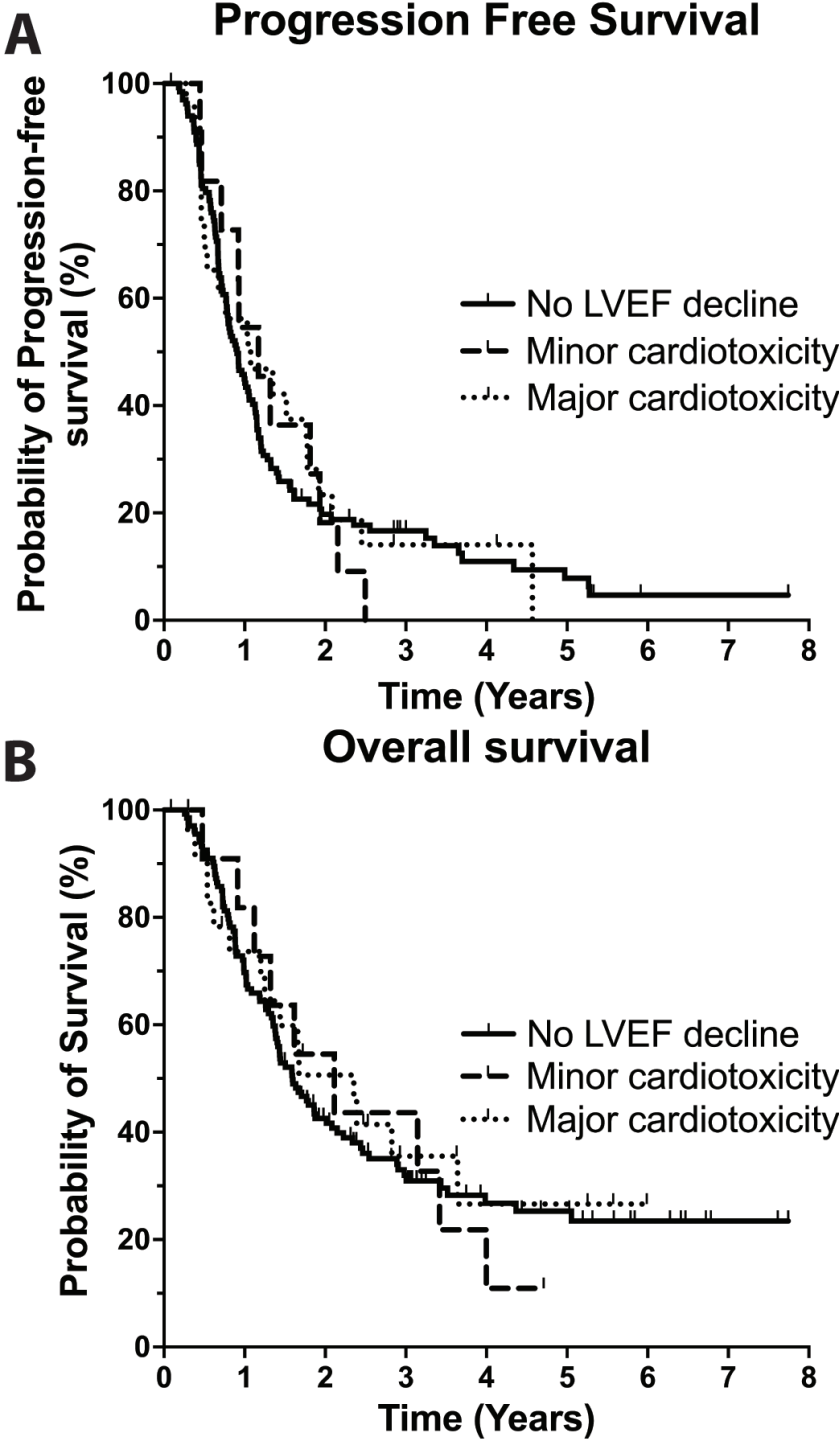
PFS and OS – Kaplan-Meier plot. Kaplan–Meier plot of progression-free survival (a) and overall survival (b) for patients with no decline in left ventricular ejection fraction (LVEF), patients experiencing minor cardiotoxicity, and patients experiencing major cardiotoxicity.

For patients with no decline in LVEF, median PFS and OS were 306 and 564 days, respectively. For patients with minor cardiotoxicity, 427 and 609 days, and for patients with major cardiotoxicity, 377 and 611 days. The Log-Rank test showed no significant difference in PFS-time and OS for patients experiencing minor cardiotoxicity (*p* = 0.9887 and *p* = 0.9440, respectively) and major cardiotoxicity (*p* = 0.7780 and *p* = 0.6961, respectively) compared to patients with no decline in LVEF (Figure 3).

## Discussion

In this retrospective analysis of an unselected Danish real-world patient cohort, 21% of melanoma patients receiving BRAFi/MEKi-therapy experienced a decline in LVEF, with 14% of these cases classified as major cardiotoxicity. No difference was found in time to LVEF decline between the use of echocardiography and MUGA-scans. The decline in LVEF was fully or partially reversible in 79% of patients following interventions, such as dose reduction, treatment pause, initiation of heart failure therapy, or continuous monitoring of LVEF without any changes in treatment.

In this study, we found a higher rate of cardiotoxicity (21%) compared to the rate (8%) reported in clinical trials [7–13]. This discrepancy may be attributed to varying definitions of cardiotoxicity [27] or the use of different imaging modalities, such as MUGA-scans and echocardiography, for assessing LVEF within the same patient cohort or across different clinical studies [28]. Notably, a similar Danish retrospective study reported a 33% rate of cardiotoxicity in a comparable cohort, which exclusively utilized MUGA-scans, in contrast to our study, which employed both MUGA-scans and echocardiography [23]. This difference in assessment methods may account for the variation in reported rates of cardiotoxicity, suggesting that MUGA-scans may have a tendency to overdiagnose cardiotoxicity in comparison to echocardiography.

Systematic assessment of cardiac dysfunction was not routinely performed using standardized scoring systems like NYHA classification. Instead, symptom documentation varied between physicians, making consistent quantification challenging. To ensure objective analysis, this study focused on LVEF-based cardiotoxicity definitions. Future studies should incorporate prospective symptom scoring for a more comprehensive evaluation.

Managing major cardiotoxicity presents a complex challenge, as it requires balancing cardiac safety with the continuation of effective antineoplastic therapy. Current expert guidelines recommend pausing MEKi treatment if there is an absolute LVEF decrease to less than 40% [29]. Importantly, the decision to discontinue MEKi for cardiac protection should be carefully weighed against its potential oncological benefits, particularly in asymptomatic cases of cardiotoxicity. In our study, 79% of patients experienced fully or partially reversible cardiac function following interventions, such as dose reduction, treatment pause, initiation of heart failure therapy, or continuous monitoring of LVEF without changes in BRAFi/MEKi treatment. Notably, 47% of patients achieved partial or full reversibility while continuing the same dosage of BRAFi/MEKi therapy. These findings suggest that LVEF decline should be managed through individualized decision-making, with repeated LVEF assessments guiding treatment adjustments.

The median time to onset of cardiotoxicity was 4.2 months with 92% of patients developing major cardiotoxicity within the 1-year evaluation, which is in line with other reports [23, 30]. No difference was observed in the time to detection between the use of echocardiography compared to MUGA-scans. These findings suggest that routine monitoring of cardiac function can be effectively performed using either modality. Additionally, routine LVEF monitoring may be safely discontinued based on clinical judgment after the 1-year evaluation, provided there are no signs of heart failure or a decline in LVEF. With a median treatment duration of 472 days and a median follow-up of 633 days, our study provides a reasonable timeframe to assess late-onset cardiotoxicity. However, longer follow-up would add valuable information to whether routine imaging can be safely discontinued after 1 year. Additionally, early disease progression often leads to treatment discontinuation and cessation of cardiac monitoring, potentially biasing the higher incidence of cardiotoxic events observed in the first year.

Patients with minor cardiotoxicity had significantly higher LVEF at baseline (70.1% ± 8.9) compared to patients with no decline in LVEF (59.8% ± 8.2). A high heart rate correlates with a high LVEF [31], and in the comparable Danish retrospective study [23], they found a similar significantly higher LVEF at baseline for patients with minor cardiotoxicity and a significant decline in heart rate simultaneously with the decline in LVEF during BRAFi/MEKi treatment. Here, it is theorized that a high metabolism caused by a high tumor load leads to an initial high heartrate [32, 33], and that the observed decreases in LVEF and heart rate are due to a reduction in tumor load resulting from the initial efficacy of BRAFi/MEKi treatment, rather than a cardiotoxic effect. Similarly, none of the patients experiencing minor cardiotoxicity developed symptoms of heart failure, initiated heart failure treatment, and underwent dose-reduction or treatment pause. All resumed MEKi-therapy without further reduction in LVEF in line with recommendations [14, 29]. This confirms that minor cardiotoxicity is not clinically significant and should not prompt changes in BRAFi/MEKi treatment but rather warrants continued monitoring of LVEF.

In this study, we found no difference in PFS and OS for patients experiencing no decline in LVEF compared to patients who experienced cardiotoxicity (major and minor). These findings align with other studies [27] but differ somewhat from a Danish retrospective study that reported significantly improved PFS and a trend toward improved OS in patients with major cardiotoxicity [23]. The discrepancy may be attributed to the relatively small sample size in the Danish study (*n* = 139), which could have influenced the results. The finding of no differences in PFS and OS between the three groups suggests that early onset of decreased LVEF may not be indicative of a poor prognosis for patients at risk of developing cardiotoxicity if it is treated adequately.

Patients with a baseline LVEF > 45% were grouped together with those having a normal LVEF. Only three patients were identified with an LVEF < 30%, making it challenging to draw definitive conclusions about the risk of treating this patient group with BRAFi/MEKi. However, this study suggests that the BRAFi/MEKi treatment is not contraindicated in patients with an LVEF < 50%, provided that heart function and symptoms of heart failure are closely monitored.

Our multivariate analysis identified baseline atrial fibrillation as a risk factor for major cardiotoxicity, while Encorafenib/Binimetinib was linked to a reduced risk. To the best of our knowledge, the association between atrial fibrillation and BRAFi/MEKi-induced cardiotoxicity is novel, with no prior reports for comparison. Given the well-documented risk of BRAFi/MEKi-related arrhythmias [29, 34, 35], baseline atrial fibrillation may have an additive effect. These findings warrant further validation but suggest heightened caution when initiating therapy in these patients. To our knowledge, no previous studies have reported a protective effect of Encorafenib/Binimetinib against cardiotoxicity, necessitating further investigation.

The potential cardiotoxic effects of immune checkpoint inhibitors (ICIs) have been increasingly recognized [29]. Given the prolonged half-life of ICIs and their broad therapeutic window, it is plausible that prior ICI therapy could contribute to an elevated risk of cardiotoxicity during subsequent BRAFi/MEKi treatment. In our patient cohort, data on prior ICI treatments, including the timing between ICI and BRAFi/MEKi therapies, have not been systematically reported. Future research should incorporate these data to elucidate the impact of prior ICI exposure on cardiotoxicity risk during targeted therapy.

This study’s strengths include real-world data reflecting clinical practice, long-term outcome analysis, and hypothesis generation for future research. However, its retrospective design, small sample size (24 major cardiotoxicity cases), and potential selection bias limit generalizability. Findings such as the association between cardiac arrhythmia and Encorafenib/Binimetinib require further validation.

In conclusion, cardiotoxicity is a notable but mostly manageable complication in patients with metastatic melanoma treated with BRAFi/MEKi, with 21% experiencing a decline in LVEF and 14% classified as major cardiotoxicity. Crucially, cardiotoxicity was reversible in 79% of patients following interventions like dose modification, treatment pauses, or heart failure therapy, allowing many to continue BRAFi/MEKi treatment without significant cardiac impairment. The identification of baseline atrial fibrillation as a significant risk factor for major cardiotoxicity highlights the need for targeted monitoring in patients with preexisting cardiac conditions. Routine LVEF monitoring during the first year of treatment remains essential, with 92% of major cardiotoxicity cases occurring within this period. However, continuous cardiac assessment beyond 1 year may not be necessary for patients without signs of cardiotoxicity. These findings suggest that individualized management of cardiotoxicity, with regular monitoring and timely intervention, can enable the safe continuation of BRAFi/MEKi therapy. Further studies are needed to investigate the underlying mechanisms of BRAFi/MEKi-induced cardiotoxicity and to improve risk stratification for optimizing patient care.

## Author contributions

JKO: Conceptualization, Data acquisition (Aarhus, Denmark), Data/Statistical Analysis, Data Visualization, Principal Contributor of Writing/Editing and Review. Read and approved final draft. IKM: Data acquisition (Odense, Denmark), Review/feedback. Read and approved final draft. LB: Data acquisition (Odense, Denmark), Review/feedback. Read and approved final draft. LMG: Review/feedback and supervision. Read and approved final draft. HS: Review/feedback and supervision. Read and approved final draft. RBF: Conceptualization, Methodology, Project administration, Legal work, Review/feedback and Supervision. Read and approved final draft.

## Supplementary Material



## Data Availability

The data that support the findings of this study are not publicly available due to information that could compromise the privacy of research participants in accordance with Danish Patient Confidentiality Laws.
