# Supplementary material for: Cardiotoxicity in patients with metastatic melanoma treated with BRAF/MEK inhibitors: a real-world analysis of incidence, risk factors, and reversibility
Source: Acta Oncol. 2025 Apr 13;64:42567. doi: 10.2340/1651-226X.2025.42567 (PMC12012651; doi:10.2340/1651-226X.2025.42567)

Supplementary material has been published as submitted. It has not been copyedited, or typeset by Acta Oncologica

Supplementary Figure 1: ROC curve for the logistic regression model as a predictor of major cardiotoxicity. AUC = 0.850, SE = 0.051, *p*-value = 5.44e-12

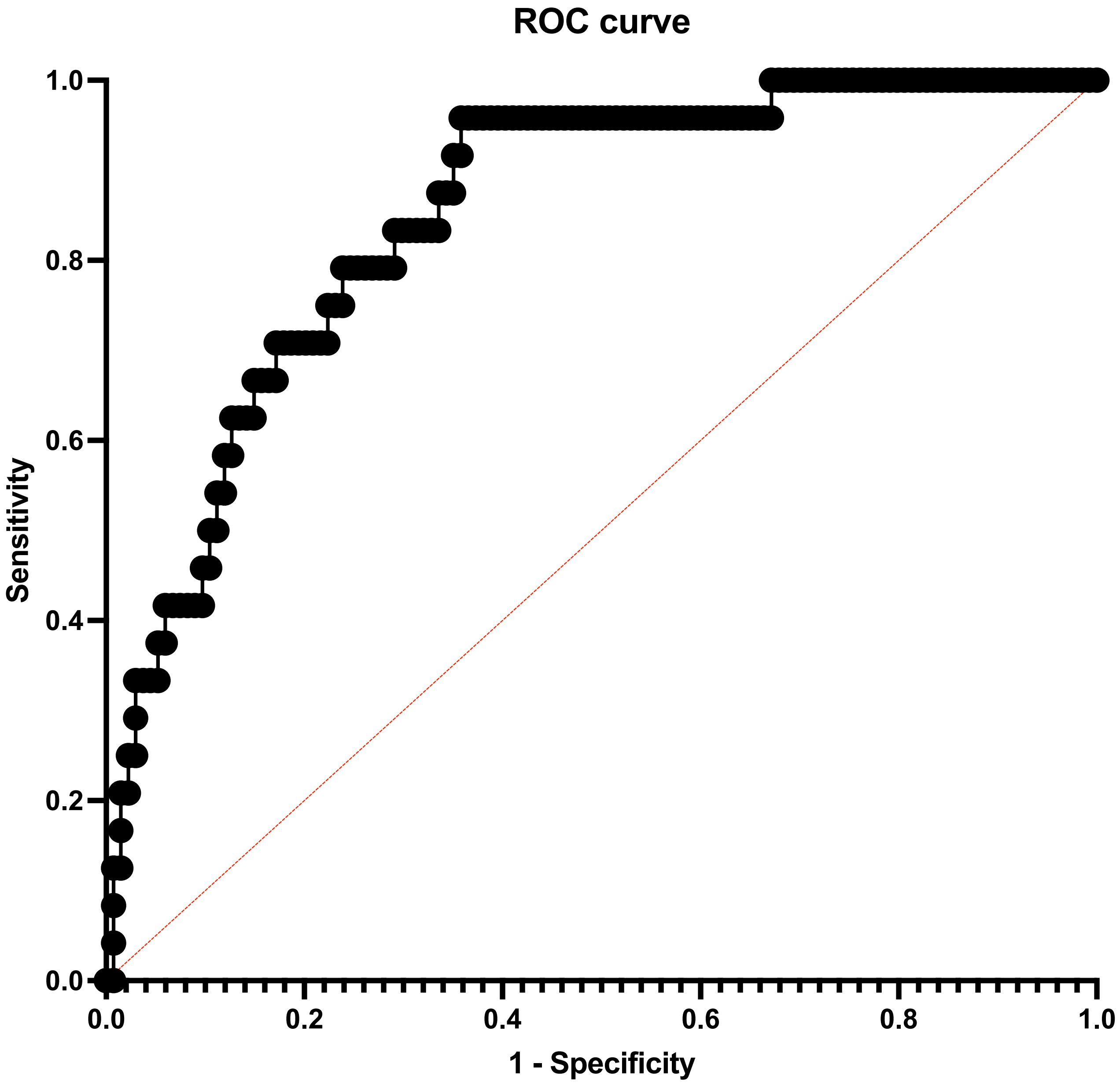

Supplement: Supplementary file 1 [file AO-64-42567-s1.pdf]
